# Two novel triazine-based quaternary ammonium salt Gemini surfactants as potential corrosion inhibitors for carbon steel in a sulfate-reducing bacteria solution: Experimental and theoretical studies

**DOI:** 10.1016/j.heliyon.2024.e40385

**Published:** 2024-11-20

**Authors:** Guofang Gao, Junxia Wang, Penghui Liang, Yilei Ruan, Dehua Wang, Li Feng, Xuemei Ma, Zhiyong Hu, Hailin Zhu

**Affiliations:** a School of Chemistry and Chemical Engineering, North University of China, Taiyuan, 030051, Shanxi, China; b Shanxi Key Laboratory of Functional Surfactants, Taiyuan, 030001, Shanxi, China

**Keywords:** Gemini surfactant, Carbon steel, Microbiological corrosion, SRB, Biocorrosion inhibitor

## Abstract

In this paper, two triazine ring–containing quaternary ammonium salt Gemini surfactants (C_12_-2-C_12_ and C_14_-2-C_14_) were synthesized. The minimum inhibitory concentrations against sulfate-reducing bacteria (SRB) of C_12_-2-C_12_, C_14_-2-C_14_ and dodecyl dimethyl benzyl ammonium chloride (1227) were determined using the double-dilution method. The performance of C_12_-2-C_12_ and C_14_-2-C_14_ in inhibiting carbon steel corrosion in the presence of SRB was examined, with 1227 serving as a control sample. The corrosion inhibition properties were assessed through static weight loss, electrochemical testing, and surface analysis. The interface adsorption behaviour of the corrosion inhibitor was explored via molecular dynamics simulations. Results indicate that the minimum inhibitory concentrations (MIC) of C_12_-2-C_12_ (0.021 mM) and C_14_-2-C_14_ (0.005 mM) are lower than that of 1227 (0.300 mM). The results of static weightlessness measurement reveal that the corrosion inhibition effects of the three surfactants on carbon steel soaked in SRB solution follow the order of C_14_-2-C_14_ > C_12_-2-C_12_ > 1227, with inhibition rates of 93.23 %, 88.45 %, and 76.49 % at a concentration of 0.2 mM, respectively. The adsorption behavior of these surfactants (1227, C_12_-2-C_12_, and C_14_-2-C_14_) on carbon steel surface in the presence of SRB conforms the Langmuir isotherm adsorption model. The outcomes of electrochemical experiments align with the static weight loss data. Furthermore, surface analysis results suggest that the surfactants can adsorb onto the carbon steel surface to form a protective film, thereby inhibiting SRB-induced corrosion.

## Highlights

• Gemini surfactant containing triazine ring can effectively inhibit the SRB corrosion.
• The effects of concentration and soaking days were investigated.
• The adsorption mechanism on carbon steel was revealed.
• C_14_-2-C_14_ has good inhibitory and bactericidal properties.

## Graphical abstract

**Figure:**
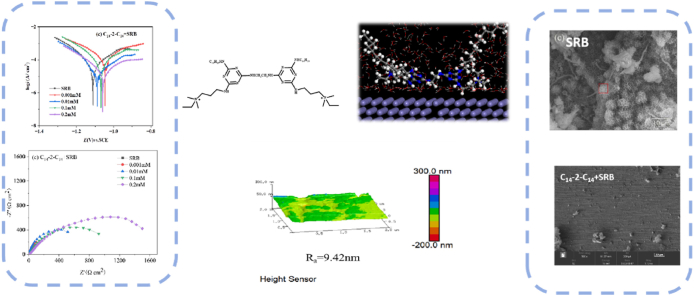

## 1 Introduction

The attachment of micro-organisms, such as sulfate-reducing bacteria (SRB), to carbon steel surfaces may induce microbiological corrosion [[1], [2], [3], [4]]. Additionally, the metabolic byproducts of such micro-organisms can exacerbate the corrosion process. For instance, the metabolic byproduct hydrogen sulfide (H_2_S) produced by SRB can result in carbon steel corrosion, leading to the creation of iron sulfide (FeS) as a corrosion product [5,6]. Consequently, it is imperative to safeguard steel from microbial corrosion to maintain the normal operation of carbon steel in the presence of micro-organisms [[7], [8], [9], [10]]. Incorporating corrosion inhibitors into the corrosion system represents a straightforward and effective protective strategy in this context [11].

Gemini surfactants have attracted widespread attention due to their excellent performance and diverse applications, which encompass sterilization, corrosion inhibition, oil displacement, dyeing assistance, and preparation of new materials [[12], [13], [14], [15], [16], [17], [18], [19], [20]]. Characterized by their high surface activity, Gemini surfactants are readily adsorbed at interfaces [[21], [22], [23]], thereby blocking metal–corrosive medium contacts, and demonstrating outstanding corrosion inhibition properties. Furthermore, Gemini surfactants containing two quaternary ammonium cations and two hydrophobic chains are efficiently adsorbed onto the negatively charged cell walls of micro-organisms, altering the permeability of these walls and resulting in potent bactericidal effects [[24], [25], [26], [27], [28], [29], [30]].

The corrosion inhibition and bactericidal properties of Gemini surfactants are correlated to their connecting group structure and hydrophobic chain structure. Labena A et al. [31] synthesized a cationic Gemini surfactant which exhibited a dual effect of sterilization and corrosion inhibition against sulfur-producing bacteria on metals in 5.49 % NaCl solution and oil field water. The sterilization effect was attributed to the interaction between the surfactant molecules and the cell membranes of sulfur-producing bacteria, while the high corrosion inhibition efficiency was linked to the surfactant molecules' strong adsorption onto metal surfaces. Pakiet et al. [32] found that quaternary ammonium Gemini surfactants were effective bactericides and corrosion inhibitors. The corrosion inhibition rate for synthesized dodecyl compounds exceeds 95 % at a low concentration of 0.018 mM. Labena A et al. discovered that Gemini surfactant with linkage group containing both phenyl and ester groups in its structure had more excellent corrosion inhibition and bactericidal properties than those with flexible or rigid linkage groups alone [33]; Gemini surfactants with amide, ester, double bond, and aromatic ring spacer groups and with dodecyl substituents exhibited higher antibacterial performance [34]. The structures and corrosion inhibition conditions for various Gemini surfactants in the references are detailed in Table 1.

**Table 1:** Gemini surfactants with different structures and corrosion inhibition conditions in the references.

number	Surfactant structure	Corrosion inhibition rates	Corrosive environment	MIC	Reference number
1		98.3 % (0.01 mM)	SRB		[18] (Our lab.)
2		–	SRB; mild steel	0.018 mM	[31]
3		95 % (1 mM)	the Hamra-sulfidogenic bacteria; mild steel		[32]
4		97 % (5 mM)	the Youmna-sulfidogenic bacterial; mild steel		[33]
5		95.5 % (5 mM)	Acidobacter ferrooxidans; mild steel		[34]

**Fig. 1:**
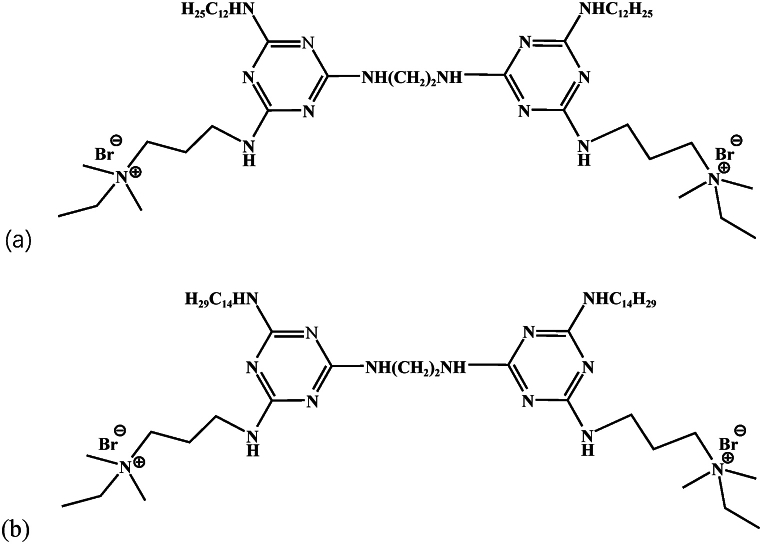
Molecular structures of C_12_-2-C_12_ and C_14_-2-C_14_: (a) C_12_-2-C_12_, (b) C_14_-2-C_14_.

The triazine ring is characterized by the presence of unpaired nitrogen heteroatoms and an abundance of π electrons, which exhibit excellent efficacy in corrosion inhibition. The bactericidal effect can be intensified by connecting two quaternary ammonium salt structures. Theoretically, quaternary ammonium salt Gemini surfactants containing triazine ring possess stronger corrosion inhibition and bactericidal properties. Therefore, three triazine-based quaternary ammonium salt Gemini surfactants with 12 hydrophobic chain length and 2 to 6 methylene linking group have been synthesized, and the effect of linking group on the corrosion inhibition in hydrochloric acid have been investigated in our previous research [35]. To further investigate the corrosion inhibition and antibacterial properties of these Gemini surfactants, this study synthesized triazine-based quaternary ammonium Gemini surfactants with 14 hydrophobic chain length and 2 methylene linking group (C_14_-2-C_14_). The synthesis process closely resembles that of the triazine-based quaternary ammonium salt Gemini surfactants with 12 hydrophobic chain length and 2 methylene linking group (C_12_-2-C_12_) as described in the literature [35]. The structures of C_12_-2-C_12_ and C_14_-2-C_14_ are shown in Fig. 1. The structural characterization spectra of C_14_-2-C_14_ are provided in the supplemental material, as shown in Figs. S1–S3. The corrosion inhibition properties of C_12_-2-C_12_ and C_14_-2-C_14_ on carbon steel in the presence of SRB were examined using static weight loss, electrochemical impedance spectroscopy, and potentiodynamic polariation. In addition, molecular dynamics (MD) simulations were conducted to study the adsorption behaviour of the corrosion inhibitor.

## 2 Material and methods

### 2.1 Reagents and instruments

Cyanuric chloride, dodecamine/tetromethamine, ethylenediamine, toluene, acetone, ethyl acetate, K_2_HPO_4_, Na_2_SO_4_, NaCl, MgSO_4_^**.**^2H_2_O, sodium lactate (C_3_H_5_O_3_Na), FeSO_4_^**.**^7H_2_O, and Na_2_S_2_O_3_, were purchased from Aladdin Reagent (Shanghai) Co. Ltd. Analytically pure ascorbic acid (C_6_H_8_O_6_), analytically pure NaCl, industrial grade NH_4_Cl, industrial grade yeast extract (C_15_H_31_N_3_O_13_P_2_), N, N-dimethyl-1,3-propanediamine, and bromoethane (C_2_H_5_Br) were purchased from Xilong Science Co. Ltd., Sinopharm Group Tianjin Co. Ltd., Hebei Chengxin Co. Ltd., and Jingxin Technology (Chengdu) Co. Ltd., respectively. Q235 carbon steel was purchased from Yangzhou Xiangwei Machinery Co. Ltd. This steel has the following elemental composition (mass fraction): C 0.14 %, S 0.13 %, Mn 0.44 %, P 0.015 %, S 0.015 %, and the rest is Fe.

An electronic balance (AL204) and a thermostatic water bath (CS 601) were obtained from METTLER Toledo Instrument (Shanghai) Co. Ltd. and Shanghai Boxun Industrial Co. Ltd., respectively. An ultra-clean workbench (SW-CJ-1D) and a biochemical incubator (SPX-80B) were obtained from the Tianjin Sedelis Experimental Analytical Instrument Factory. An autoclave (YXQ-LS-18SI), a pH meter (PHS-3E), and an atomic force microscope (Bruker Dimension ICON) were obtained from Shanghai Boxun Industrial Co. Ltd., Shanghai Yi Electrical Scientific Instrument Co. Ltd., and Bruker GMBH of Germany, respectively.

### 2.2 Strain source and culture medium preparation

The SRB used in this experiment was obtained from the China General Microbial Culture Collection Center (CGMCC number 1.3469).

The chemicals in solution A presented in Table 2 were dissolved in a certain amount of distilled water, subsequently boiled and cooled. The pH was adjusted to 7.8 using 1 mol/L NaOH. The medium was deoxygenated with nitrogen gas for 2–3 min, and sterilized at an elevated temperature (121 °C) for 20 min. Component B was sterilized on a sterile operating table via UV irradiation for a span of 30 min. Then, it was introduced to Solution A, thoroughly agitated, and subsequently set aside.

**Table 2:** Culture medium composition.

Composition(Solution A)	content(g L^−1^)	composition(Solution B)	content(g L^−1^)
K_2_HPO_4_	0.5	FeSO_4_^**.**^7H_2_O	0.5
NH_4_Cl	1.0	Sodium thioglycolate	0.1
Na_2_SO_4_	1.0	Vitamin C	0.1
CaCl_2_^**.**^2H_2_O	0.1		
MgSO_4_^**.**^2H_2_O	2.0		
Dl-sodium lactate	2.0		
Yeast extract	1.0		

### 2.3 Culture and activation of SRB

Upon thawing the strains, the bacterial solution was transferred to an anaerobic tube containing the medium, deoxygenated with nitrogen for 2–3 min, and then cultivated in a biochemical incubator at 30 °C for 1–2 d. The strains were successfully activated if the liquid in the anaerobic tube turned black; otherwise, they will be reactivated.

### 2.4 Growth curve measurement

The change curve of SRB number with incubation time was measured according to the national standard GB/T 14643.5–2009, titled ‘Determination Method of Bacterial Algae in Industrial Circulating Cooling Water Part 5: Determination of Sulfate-Reducing Bacteria MPN Method’.

### 2.5 Minimum inhibition concentration

The minimum inhibitory concentration (MIC) was determined using the double-dilution method. The surfactant was diluted into a series of concentration gradient solutions with culture medium and added to the test tubes separately, with three parallel test tubes set for each concentration. A 2 % SRB bacterial solution was added to the test tubes and incubated in a biochemical incubator at 30 °C for 14 d. Following this period, the color alteration of the medium within the test tubes was meticulously documented. When three tubes did not turn black and all subsequent tubes did not turn black, the corresponding concentration was determined as its MIC.

### 2.6 Weight loss measurements

Weight loss measurements were conducted by immersing carbon steel sheets in solutions containing SRB without and with different surfactant concentrations (1227/C_12_-2-C_12_/C_14_-2-C_14_) for 21 d. After being soaked, the Q235 carbon steel samples were treated with a solution consisting of hydrochloric acid, deionized water, and hexamethylenetetramine to eliminate biofilm and corrosion products. Subsequently, the samples were rinsed with distilled water and anhydrous ethanol, dried, and weighed.

### 2.7 Electrochemical tests

Electrochemical tests were carried out on the test system of the Electrochemical Workstation (CS2350M). Platinum electrodes and saturated glycury electrodes were used as counter electrodes and reference electrodes, respectively. The Q235 carbon steel specimens, which were sealed with epoxy resin as the working electrode, had an area of 1 cm × 1 cm. The working electrode was exposed to UV light for at least 30 min before use to ensure that it would not be contaminated by other bacteria. The anaerobic flasks were filled with different surfactant concentrations and a 2 % bacterial solution, except for the blank control group. Each anaerobic flask was ventilated with N_2_ for 5 min to exclude O_2_ before sealing. The test solutions were placed in an incubator and left to cultivate at 30 °C (±1 °C) for certain durations (1, 3, 5, 10, 15, and 21 d). The open-circuit potential was first detected. After it was stabilized, a sinusoidal potential perturbation of 5 mV was applied across a frequency range of 10^−2^ –10^5^ Hz to perform electrochemical impedance spectroscopy (EIS) for the different immersion periods. The impedance data were then analyzed using Zsimpwin 330. Following a 21-day immersion period, the kinetic potential polarization curves of the carbon steel sheets were measured at a scan rate of 0.2 mV s^−1^. Various electrochemical parameters were subsequently calculated using Tafel extrapolation.

### 2.8 Surface measurements

The surfaces of carbon steel sheets were analyzed through scanning electron microscopy (SEM), atomic force microscopy (AFM), and X-ray photoelectron spectroscopy (XPS) after 21 d of immersion in SRB-containing solutions without and with different concentrations of surfactants (1227/C_12_-2-C_12_/C_14_-2-C_14_).

### 2.9 MD analysis

The simulations were performed using the Forcite module in the Materials Studio 8.0 software. The simulation process was conducted in a three-dimensional box (32.27 Å × 32.27 Å × 14.27 Å) using an iron (110) surface as the reference surface. Approximately 500H_2_O and 1 corrosion inhibitor molecules were added in the iron box. The COMPASS forcefield and NVT ensemble were utilized for the simulations. The time step, total simulation time and temperature were 0.1 fs, 5000 ps and 30 °C, respectively.

## 3 Results and discussion

### 3.1 Growth curve

To better study the effect of SRB on the corrosion of carbon steel, the growth curve of SRB was investigated. The grow curve are presented in Fig. S4.

As shown in Fig. S4, the growth curve is divided into three phases. SRBs grew exponentially within 1–4 d (logarithmic phase). This phase is logarithmic, which can be attributed to the presence of sufficient nutrients in the medium resulting in a very active SRB growth [[36], [37], [38], [39]] and rapid proliferation by two divisions. The trend of increase in SRB number slows down from 4 to 7 d (stationary phase) and reaches a peak on day 7 (1.8 × 10^8^ cells mL^−1^). A number of SRBs decay after 7 d (decline phase) due to nutrient depletion and metabolite accumulation, while the lack of nutrients in the medium results in faster cell death [40,41]. Moreover, active cells almost disappear during the decay stage.

### 3.2 Minimum inhibitory concentration

The experimental results show that the lowest inhibitory concentrations of 1227, C_12_-2-C_12_ and C_14_-2-C_14_ are 0.300, 0.021, and 0.005 mM, respectively. The Gemini surfactant containing triazine ring quaternary ammonium salt with hydrophobic chain of 14 has the lowest inhibitory concentration.

### 3.3 Static weightlessness analysis

**Fig. 2:**
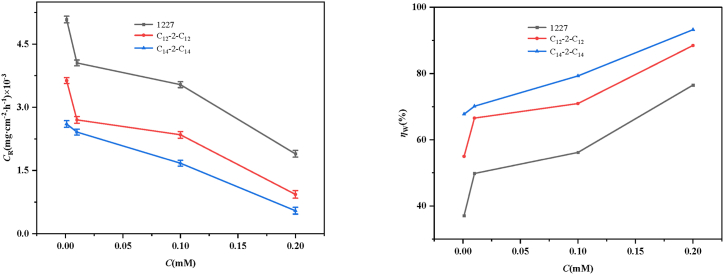
Corrosion inhibition of carbon steel after 21 d immersion in SRB-containing medium solutions with different concentrations of 1227, C_12_-2-C_12_ and C_14_-2-C_14_.

The protective effect of different concentrations of 1227/C_12_-2-C_12_/C_14_-2-C_14_ in SRB-containing solutions on Q235 carbon steel was investigated through static weight loss (Fig. 2). The corrosion rate and inhibition rate data are presented in Table 3.

**Table 3:** Corrosion rate and corrosion inhibition data.

Inhibitor	C (mM)	*C*_R_ (mg cm^−2^ h^−1^)	*θ*	*η*_W_ (%)
SRB		0.0081 ± 0.00007	–	–
1227	0.001	0.0051 ± 0.00008	0.37	37.05
	0.01	0.0041 ± 0.00007	0.50	49.80
	0.1	0.0035 ± 0.00007	0.56	56.18
	0.2	0.0019 ± 0.00008	0.76	76.49
C_12_-2-C_12_	0.001	0.0036 ± 0.00007	0.55	54.98
	0.01	0.0027 ± 0.00008	0.67	66.53
	0.1	0.0023 ± 0.00008	0.71	70.92
	0.2	0.0009±±0.00009	0.88	88.45
C_14_-2-C_14_	0.001	0.0026±±0.00008	0.68	67.73
	0.01	0.0024±±0.00007	0.70	70.12
	0.1	0.0017±±0.00007	0.79	79.28
	0.2	0.0005±±0.00008	0.93	93.23

As shown in Fig. 2, with the increase of 1227/C_12_-2-C_12_/C_14_-2-C_14_ concentration, the corrosion rate is significantly reduced, and the inhibition efficiency is significantly improved, demonstrating the strong inhibitive properties of the surfactants [42]. This is because higher concentrations of surfactant lead to greater adsorption capacity, resulting in fewer available sites for adsorbing corrosive elements. From Tables 3 and it can be seen that carbon steel has the highest corrosion rate in SRB medium, which may be due to the inherent bacterial molecules inducing corrosion through anodic dissolution, resulting in significant loss of carbon steel quality. When 1227, C_12_-2-C_12_ and C_14_-2-C_14_ were added, the corrosion rate decreased, indicating effective corrosion inhibition after the addition of surfactants. When the concentration is 0.2 mM, the corrosion inhibition rates of the three surfactants are 76.49 %, 88.45 %, and 93.23 %, respectively. Compared with 1227, C_12_-2-C_12_ can further reduce the corrosion rate of carbon steel in SRB solution. This is mainly due to the negative attraction of N^+^ and SRB, which changes the permeability of bacterial cell walls and causes cell death. Double quaternary ammonium salts are more effective in inhibiting the corrosion of carbon steel in SRB containing solutions than single quaternary ammonium salts [18]. Compared with C_12_-2-C_12_, C_14_-2-C_14_ has a higher corrosion inhibition rate in SRB solution, mainly due to its surface coverage increases with the increase of hydrophobic chain length. In addition, the bactericidal effects of quaternary ammonium salt surfactants may enhance the corrosion inhibition [43]. The minimum inhibitory concentration of C_12_-2-C_12_ (0.021 mM) is much lower than that of 1227 (0.300 mM), which has a better bactericidal effect, leading to better corrosion inhibition. Moreover, the bactericidal performance of the corrosion inhibitor with the hydrophobic chain length of 14 is best within the research condition, with the minimum inhibitory concentration of 0.005 mM, showing the best corrosion inhibition.

### 3.4 Electrochemical analysis

#### *3.4.1* *Effect of the concentration on inhibition and sterilization of 1227*/*C*_*12*_*-2-C*_*12*_/*C*_*14*_*-2-C*_*14*_

(1) Dynamic potential polarization

**Fig. 3:**
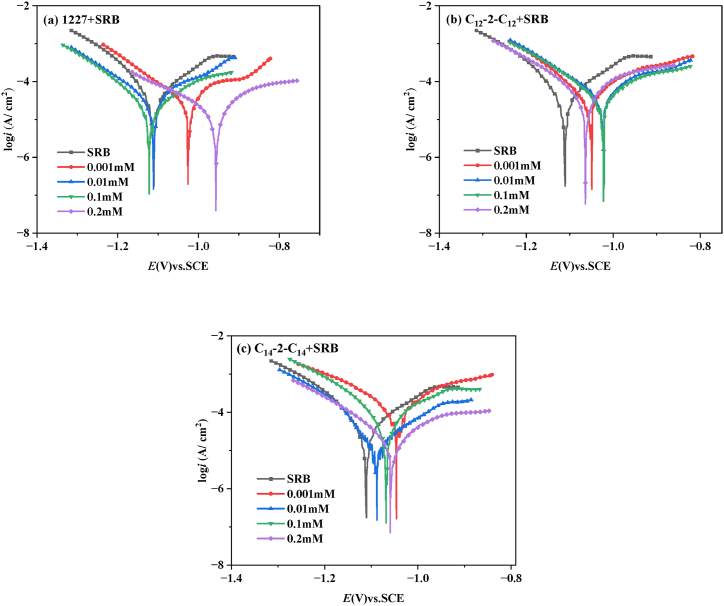
Kinetic potential polarization diagram of carbon steel immersed in SRB solution with different concentrations of 1227、C_12_-2-C_12_、C_14_-2-C_14_ for 21d.

Fig. 3 shows the potentiodynamic polarization curves of Q235 carbon steel immersed in SRB solutions containing different concentrations of 1227, C_12_-2-C_12_, and C_14_-2-C_14_ for 21 d. The corresponding fitting parameters by using Tafel extrapolation are collected in Table 4. As shown in Fig. 3, compared with the SRB solution without corrosion inhibitors, the polarization curve of the SRB solution with 1227, C_12_-2-C_12_ or C_14_-2-C_14_ shifted towards lower current density, indicating significant inhibitory effect of the three surfactants [44]. As the concentration of 1227, C_12_-2-C_12_ or C_14_-2-C_14_ increase, the corrosion current density gradually decreases, thereby reducing the corrosion rate. This indicates that the addition of 1227, C_12_-2-C_12_ or C_14_-2-C_14_ slows down the corrosion of SRB on carbon steel.

**Table 4:** Fitting data of dynamic potential polarization.

	C (mM)	*E*_corr_(V)vs.SCE	*i*_corr_ (μA cm^−2^)	*b*_*a*_ (V dec^−1^)	*b*_c_ (V dec^−1^)	*η*_pp_ (%)
SRB		−1.104 ± 0.009	85.92 ± 0.23	0.203 ± 0.003	−0.145 ± 0.007	–
1227	0.001	−1.014 ± 0.010	54.75 ± 0.56	0.506 ± 0.006	−0.113 ± 0.004	36.28
	0.01	−1.116 ± 0.006	38.50 ± 0.38	0.269 ± 0.005	−0.097 ± 0.008	55.19
	0.1	−1.120 ± 0.004	30.90 ± 0.30	0.150 ± 0.006	−0.145 ± 0.004	64.04
	0.2	−0.953 ± 0.011	21.64 ± 0.40	0.183 ± 0.008	−0.262 ± 0.005	74.81
C_12_-2-C_12_	0.001	−1.063 ± 0.008	49.91 ± 0.12	0.041 ± 0.003	−0.094 ± 0.006	41.91
	0.01	−1.023 ± 0.006	29.51 ± 0.36	0.135 ± 0.003	−0.066 ± 0.007	65.65
	0.1	−1.015 ± 0.014	26.82 ± 0.48	0.095 ± 0.006	−0.115 ± 0.009	68.78
	0.2	−1.078 ± 0.010	18.34 ± 0.55	0.046 ± 0.004	−0.099 ± 0.005	78.65
C_14_-2-C_14_	0.001	−1.035 ± 0.003	39.36 ± 0.27	0.166 ± 0.004	−0.110 ± 0.002	54.19
	0.01	−1.079 ± 0.015	24.96 ± 0.15	0.160 ± 0.005	−0.112 ± 0.009	70.95
	0.1	−1.065 ± 0.009	23.91 ± 0.19	0.183 ± 0.001	−0.127 ± 0.006	72.17
	0.2	−1.046 ± 0.006	12.91 ± 0.43	0.008 ± 0.006	−0.111 ± 0.007	84.97

As can be seen from Table 4, the corrosion current density in SRB solution without corrosion inhibitors is 85.92 μA cm^−2^. Upon the addition of 0.2 mM C_14_-2-C_14_, the corrosion current density decreases to 12.91 μA cm^−2^. The decrease in corrosion current density with addition of surfactant and the significant increase in inhibition efficiency are attributed to the fact that the surfactant molecules can be adsorbed on the carbon steel surface, hindering the further corrosion of SRB. When the addition concentration is 0.2 mM, the corrosion inhibition rates of the three surfactants are 74.81 %, 78.65 %, and 84.97 %, respectively. It shows that C_14_-2-C_14_ has the best corrosion inhibition effect. Furthermore, the positive shift in the corrosion potential (*E*_corr_), being less than 85 mV, indicating that the surfactants are mixed-type corrosion inhibitor mainly used for anode control.

(2) Electrochemical impedance

**Fig. 4:**
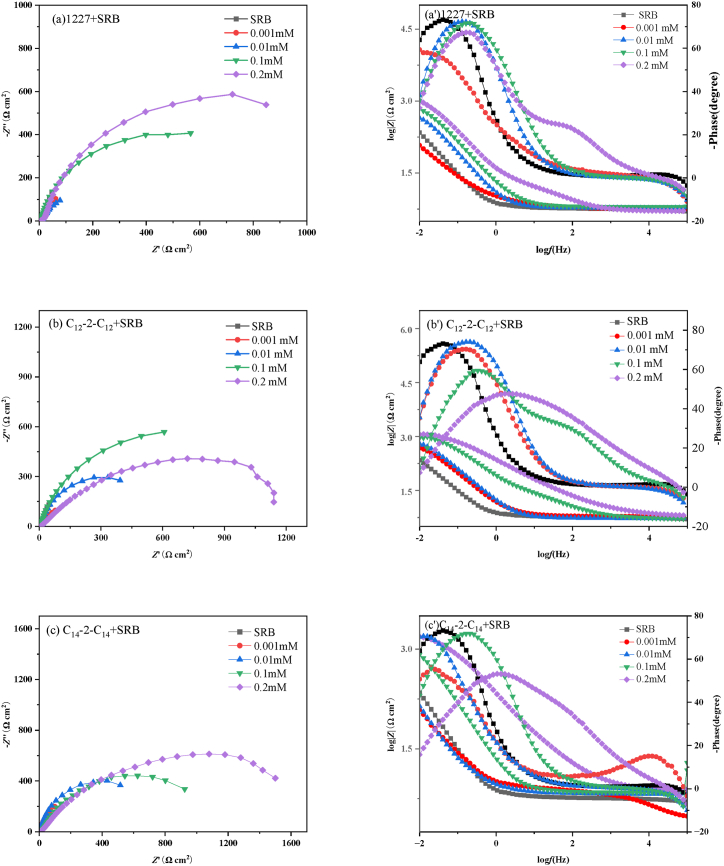
Nyquist (a、b、c) and Bode (a'、b'、c') plots of carbon steel immersed in SRB solutions with different concentrations of 1227、C_12_-2-C_12_、C_14_-2-C_14_ for 21 d.

Fig. 4 shows the Nyquist and Bode plots of Q235 carbon steel immersed in SRB solutions with different concentrations of 1227, C_12_-2-C_12_, and C_14_-2-C_14_ for 21 d.

As shown in Fig. 4, the Nyquist plot presents a slender arc, which may be due to the surface roughness of the electrode and the non-uniformity of frequency dispersion. The bigger the impedance of corrosion, the better corrosion inhibition property that the corrosion inhibitor has [45]. The impedance arc diameter after adding 1227, C_12_-2-C_12_, and C_14_-2-C_14_ is larger than that in SRB medium without inhibitor addition, indicating better corrosion inhibition of three surfactants. Moreover, the diameter of the semicircle significantly increases with increasing concentrations of 1227, C_12_-2-C_12_, and C_14_-2-C_14_. When the concentration is the same, the impedance arc diameter of C_14_-2-C_14_ is larger than that of 1227 and C_12_-2-C_12_, indicating that C_14_-2-C_14_ has the highest inhibition efficiency. This may be due to the surfactant can form an adsorption film on the surface of the steel (electrode), thereby hindering the corrosion element contact the steel and inhibiting the corrosion.

The EIS data are fitted using the equivalent circuit shown in Fig. S6 (a). *R*_S_ is the solution resistance, *R*_f_ is the membrane resistance, *R*_ct_ is the charge transfer resistance, *CPE*_f_ is the membrane capacitance, and *CPE*_dl_ is the double layer capacitance. The impedance value of the constant phase element (CPE), the *C*_dl_ value of the double layer capacitor, and the retardation rate (*η*_eis_) were calculated according to Eq. (1) ∼ (4), and the results were listed in Table 5.

(1) $$Z_{\text{CPE}}=\frac{1}{Y_{0}{\left(j\omega \right)}^{n}}$$

where *Z*_CPE_ and *Y*_0_ are the impedance and admittance of CPE, respectively, *j* is the virtual root (i.e. $j=\sqrt{-1}$), *ω* is the angular frequency, *n* is the dispersion effect index of CPE.

(2) Cdl=Y01n(RsRctRs+Rct)1−nn

(3) $$C_{f}=Y_{0}{\left(2\pi f_{\max}\right)}^{n-1}$$

(4) $$\eta_{\text{eis}}=\frac{R_{P}-{R_{P}}^{0}}{R_{P}}\times 100$$

where *R*_p_^0^ and *R*_p_ are the corrosion resistance in the solution without and with surfactants, respectively.

**Table 5:** Fitted data of electrochemical impedance spectrum.

Inhibitor	*C* (mM)	*R*_s_ (Ω cm^2^)	*C*_f_ (mF cm^−2^)	n_1_	*R*_f_ (Ω cm^2^)	*C*_dl_ (mF cm^−2^)	n_2_	*R*_ct_ (Ω·cm^2^)	*R*_p_ (Ω·cm^2^)	*η*_eis_ (%)
SRB	–	5.31 ± 0.03	3.57 ± 0.14	0.80	7.39 ± 0.52	5.54 ± 0.20	0.80	411.00 ± 1.43	418.38	–
1227	0.001	5.70 ± 0.02	5.12 ± 0.21	0.95	10.12 ± 0.39	13.25 ± 0.36	0.91	853.75 ± 3.49	863.87	51.57
	0.01	6.72 ± 0.01	4.98 ± 0.16	0.88	15.13 ± 0.76	5.26 ± 0.41	0.89	963.54 ± 10.37	978.67	57.25
	0.1	6.33 ± 0.03	5.25 ± 0.25	0.94	20.52 ± 0.22	4.26 ± 0.27	0.96	1235.50 ± 15.50	1256.02	66.69
	0.2	5.83 ± 0.02	2.14 ± 0.13	0.79	15.91 ± 0.27	6.50 ± 0.19	0.87	1680.00 ± 11.49	1695.91	75.33
C_12_-2-C_12_	0.001	2.92 ± 0.04	10.23 ± 0.26	0.99	7.33 ± 0.46	10.69 ± 0.50	0.91	1253.99 ± 9.74	1261.32	66.83
	0.01	6.24 ± 0.03	8.89 ± 0.15	0.98	18.23 ± 0.25	7.19 ± 0.34	0.81	1609.07 ± 12.68	1627.30	74.29
	0.1	7.83 ± 0.01	5.56 ± 0.19	0.94	20.05 ± 0.42	8.55 ± 0.17	0.85	3065.35 ± 16.95	3085.40	86.44
	0.2	6.65 ± 0.02	6.88 ± 0.17	0.67	33.14 ± 0.30	0.88 ± 0.28	0.63	3515.46 ± 11.06	3548.60	88.21
C_14_-2-C_14_	0.001	5.70 ± 0.03	23.26 ± 0.28	0.96	4.88 ± 0.37	9.23 ± 0.66	0.92	1328.39 ± 7.48	1333.27	68.62
	0.01	6.72 ± 0.02	15.23 ± 0.12	0.94	15.32 ± 0.45	8.23 ± 0.31	0.89	2210.11 ± 12.42	2225.43	81.20
	0.1	7.53 ± 0.01	9.89 ± 0.15	0.98	21.26 ± 0.67	7.89 ± 0.21	0.92	3450.77 ± 8.71	3472.03	87.95
	0.2	7.62 ± 0.03	3.29 ± 0.16	0.65	84.60 ± 0.38	7.47 ± 0.44	0.76	4142.00 ± 10.05	4226.60	90.10

As shown in Table 5, compared with the SRB medium, the addition of surfactants can increase the value of charge transfer resistance (*R*_ct_). Moreover, the corrosion inhibition rate also increases as the surfactant concentration increases, indicating that the presence of surfactants can inhibit the corrosion of carbon steel by SRB to a certain extent. When the concentration is 0.2 mM, the corrosion inhibition rate of 1227, C_12_-2- C_12_, and C_14_-2-C_14_ is 75.33 %, 88.21 %, and 90.10 %, respectively. The corrosion inhibition rate of C_12_-2-C_12_ is higher than that of 1227, indicating that the double R_4_N^+^ group can improve the corrosion inhibition and bactericidal properties of surfactants. The corrosion inhibition of C_14_-2-C_14_ is better than that of C_12_-2-C_12_, indicating that the increase of the hydrophobic chain length will change the permeability of the cell membrane and lead to the death of SRB.

#### 3.4.2 Effect of soaking days on the adsorption behavior of 1227/C_12_-2-C_12_/C_14_-2-C_14_

(1) Electrochemical impedance

**Fig. 5:**
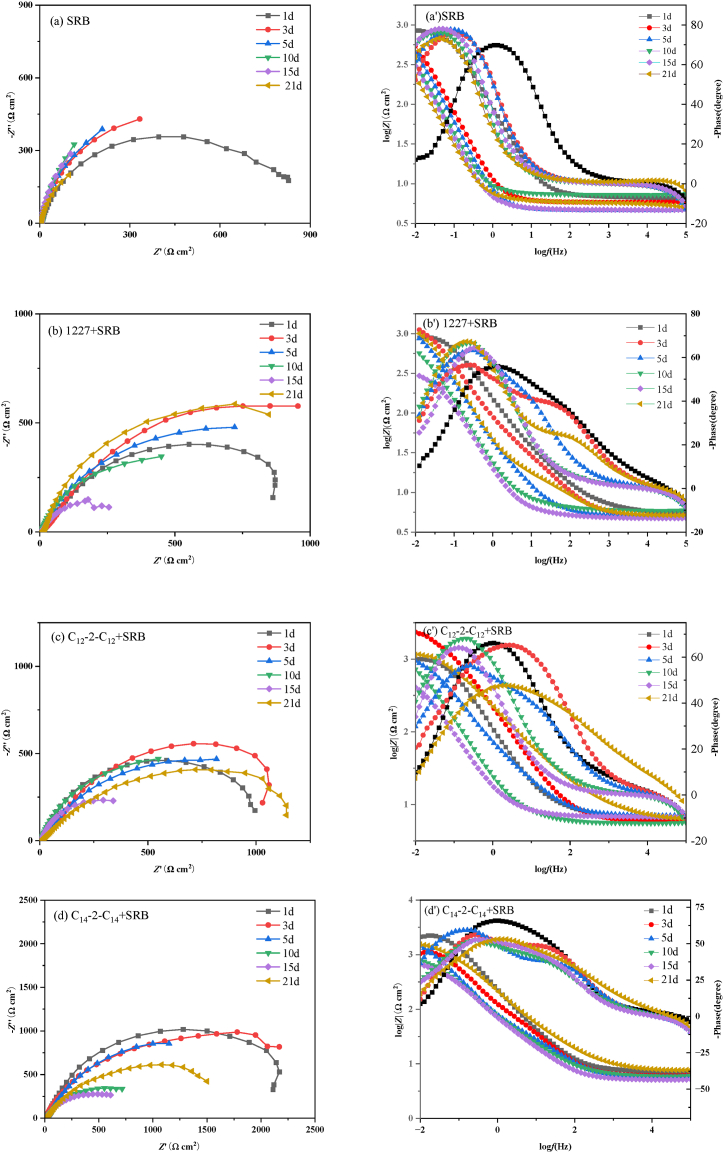
Nyquist (a、b、c、d) and Bode (a'、b'、c'、d') plots of carbon steel immersed in SRB solutions with 1227、C_12_-2-C_12_、C_14_-2-C_14_ for different days.

Fig. 5 shows the Nyquist and Bode plots for Q235 carbon steel immersed in SRB solutions of 0.2 mM 1227, C_12_-2-C_12_, and C_14_-2-C_14_ for different days. The EIS data were fitted using the equivalent circuit shown in Fig. S6 (a) and (b). Fig. S6 (b) was used for carbon steel immersed in SRB solution for 1 day, and Fig. S6 (a) was used to analyze other impedance plots. W represents the Warburg impedance. The fitted data are presented in Table 6.

**Table 6:** Electrochemical impedance spectrum fitting data.

Inhibitor	Day	*R*_s_ (Ω cm^2^)	*C*_f_ (mF cm^−2^)	n_1_	*R*_f_ (Ω cm^2^)	*C*_dl_ (mF cm^−2^)	n_2_	*R*_ct_ (Ω cm^2^)	*R*_p_ (Ω cm^2^)	*η*_eis_ (%)
SRB	1	6.73 ± 0.03	1.39 ± 0.07	0.92	3.70 ± 0.14	6.76 ± 0.78	0.90	838.10 ± 3.50	841.80	–
	3	5.87 ± 0.01	10.61 ± 0.06	1.00	2.55 ± 0.11	13.05 ± 0.34	0.87	774.00 ± 2.48	799.45	–
	5	4.64 ± 0.03	14.01 ± 0.10	1.00	1.48 ± 0.09	7.50 ± 0.28	0.88	845.00 ± 4.27	846.48	–
	10	7.29 ± 0.02	34.50 ± 0.09	0.91	4.74 ± 0.17	9.33 ± 0.33	1.00	369.00 ± 1.93	373.74	–
	15	4.71 ± 0.01	38.01 ± 0.12	0.92	4.53 ± 0.13	6.73 ± 0.26	1.00	433.00 ± 3.46	437.53	–
	21	5.31 ± 0.03	3.57 ± 0.14	0.80	7.39 ± 0.52	5.54 ± 0.20	0.80	411.00 ± 1.43	418.38	–
1227	1	5.75 ± 0.02	5.30 ± 0.06	0.70	48.24 ± 0.24	5.64 ± 0.38	0.81	991.00 ± 4.82	1039.24	19.00
	3	5.05 ± 0.03	3.03 ± 0.07	0.72	53.25 ± 0.27	3.94 ± 0.18	0.76	1574.00 ± 6.27	1627.25	50.87
	5	5.06 ± 0.02	7.08 ± 0.05	0.76	69.91 ± 0.19	4.46 ± 0.20	0.95	2738.00 ± 9.53	2807.91	69.85
	10	5.91 ± 0.02	7.22 ± 0.08	0.84	16.35 ± 0.34	6.71 ± 0.31	0.85	830.50 ± 7.28	1046.85	64.30
	15	4.78 ± 0.01	7.92 ± 0.06	0.84	29.00 ± 0.21	6.03 ± 0.14	0.86	1552.60 ± 6.31	1581.60	72.34
	21	5.83 ± 0.02	2.14 ± 0.13	0.79	15.91 ± 0.27	6.50 ± 0.19	0.87	1680.00 ± 11.49	1695.91	75.33
C_12_-2 -C_12_	1	6.15 ± 0.03	6.64 ± 0.04	0.84	156.90 ± 0.56	5.53 ± 0.16	0.84	1136.00 ± 6.87	1292.90	35.18
	3	5.97 ± 0.01	5.69 ± 0.07	0.78	34.00 ± 0.17	6.26 ± 0.39	1.00	1814.50 ± 2.36	1848.50	56.75
	5	6.95 ± 0.02	7.88 ± 0.03	0.68	160.40 ± 0.46	6.16 ± 0.40	0.92	3660.00 ± 8.51	3820.40	77.84
	10	5.59 ± 0.02	6.70 ± 0.04	0.85	5.58 ± 0.23	8.02 ± 0.15	0.84	1609.00 ± 4.78	1614.58	76.85
	15	6.84 ± 0.03	7.67 ± 0.06	0.85	9.68 ± 0.19	11.02 ± 0.28	0.86	1991.50 ± 2.99	2001.18	78.14
	21	6.65 ± 0.02	6.88 ± 0.17	0.67	33.14 ± 0.30	0.88 ± 0.28	0.63	3515.46 ± 11.06	3548.60	88.21
C_14_-2 -C_14_	1	7.23 ± 0.03	7.78 ± 0.05	0.82	8.00 ± 0.18	3.73 ± 0.13	0.90	1435.00 ± 5.38	1443.00	41.66
	3	6.03 ± 0.02	7.12 ± 0.07	0.75	153.30 ± 0.35	1.38 ± 0.11	0.86	1819.00 ± 5.72	1972.30	59.47
	5	5.73 ± 0.04	2.50 ± 0.06	0.74	477.40 ± 0.41	4.39 ± 0.20	0.78	4083.00 ± 6.54	4560.40	81.44
	10	5.90 ± 0.01	6.12 ± 0.08	0.65	131.40 ± 0.27	1.60 ± 0.14	0.87	1727.00 ± 5.56	1858.40	79.89
	15	5.11 ± 0.03	5.28 ± 0.04	0.77	32.43 ± 0.18	5.03 ± 0.10	0.67	2872.70 ± 4.66	2905.13	84.94
	21	7.62 ± 0.03	3.29 ± 0.16	0.65	84.60 ± 0.38	7.47 ± 0.44	0.76	4142.00 ± 10.05	4226.60	90.10

**Fig. 6:**
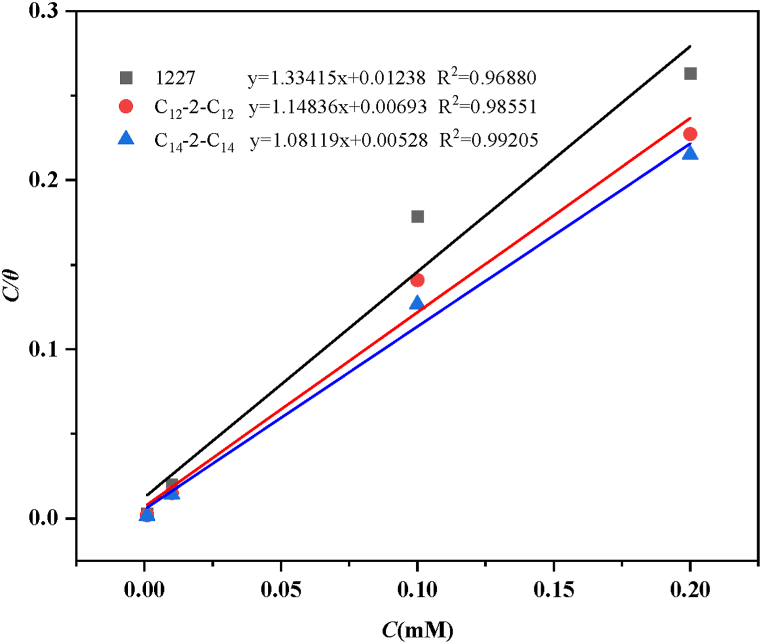
Langmuir isothermal adsorption model fitting of carbon steel in SRB-containing medium solutions of 1227, C_12_-2-C_12_ and C_14_-2-C_14_.

As shown in Fig. 5, the impedance arc diameter of the surfactant system increases with increasing immersion time, reaches its peak after 5 d, and then decreases (see Table 6). The increase in the impedance arc diameter is caused by the gradual formation of dense corrosion product film and SRB biofilm [46,47]. In the SRB solution without a surfactant, the change in *R*_P_ is caused by the corrosion of Q235 carbon steel by bacterial activity [[48], [49], [50]]. Among the three surfactants, C_14_-2-C_14_ has the largest semi-circular diameter, indicating its superior corrosion inhibition effect. According to the determination of the minimum inhibitory concentration of the three corrosion inhibitors in Section 3.3, it can be concluded that C_14_-2-C_14_ not only has a good bactericidal and bacteriostatic effect on SRB but also forms a dense adsorption film at the interface, indicating a good corrosion inhibition effect [51,52]. As shown in Fig. 6 (b)–6 (d), the impedance arc semicircle diameter with addition of surfactants is larger than that with the addition of SRB only, indicating that all three corrosion inhibitors can effectively inhibit carbon steel corrosion against micro-organisms.

As shown in Table 6, *R*_ct_ increases as increasing soaking time, which may be attributed to the low metabolic activity of SRB, the increase thickness of biofilm and corrosion product layer, and the increase adsorption of surfactant molecule on the carbon steel surface [53,54]. From 5 to 10 d, *R*_ct_ decreases, and high concentrations of corrosive metabolites (sulfides and organic acids) cause the increase in corrosion rate [55,56]. From 10 to 21 d, *R*_ct_ increases owing to a reduction in the number of SRB and the increase in the thickness of surfactant adsorption film on the carbon steel surface, playing a notable role in inhibition. The corrosion inhibition rate of C_12_-2-C_12_ is higher than that of 1227, indicating that the dual R_4_N^+^ group can effectively slow down the corrosion of SRB on carbon steel. This is mainly because the R_4_N^+^ group can change the permeability of cells and cause cell death, playing a bactericidal role. Compared with C_12_-2-C_12_, adding C_14_-2-C_14_ leads to a better corrosion inhibition effect, indicating that the length of hydrophobic chains affects the corrosion inhibition and bactericidal performance of surfactants.

### 3.5 Adsorption isotherm

Adsorption isotherms can be used to evaluate the performance of inhibitors. The Langmuir isotherm model is fitted based on the static weight loss results.

(5) $$\frac{C}{\theta }=C+\frac{1}{K_{\text{ads}}}$$

Where *K*_ads_ is the adsorption equilibrium constant, *C* is the concentration of corrosion inhibitor, and *θ* is the coverage of the metal surface (Table 3). The fitting results are shown in Fig. 6. As can be seen from Fig. 6, the three surfactants (1227, C_12_-2-C_12_, and C_14_-2-C_14_) all conform well to the Langmuir isotherm adsorption line.

The *ΔG*_ads_ can be calculated from K_ads_ by Eq. (6) as below.

(6) $$\Delta G_{\text{ads}}=‐RT\;\ln\left(55.5K_{\text{ads}}\right)$$

Where 55.5 is the molar concentration of water. The obtained results are presented in Table 7.

**Table 7:** Adsorption eigenvalues of corrosion inhibitors.

Inhibitor	*R*^2^	Slope	K_ads_	*ΔG*_ads_ (kJ mol^−1^)
1227	0.96880	1.33415	80.76	−21.18
C_12_-2-C_12_	0.98551	1.14836	144.30	−22.64
C_14_-2-C_14_	0.99205	1.08119	189.39	−23.33

One can see from Table 7, the negative value of *ΔG*_ads_ indicates that the adsorption of the surfactant on carbon steel is a spontaneous process. Furthermore, the absolute *ΔG*_ads_ value of the corrosion inhibitors ranges from 20 to 40 kJ mol^−1^, indicating a mixed adsorption type.

### 3.6 Surface analysis

#### 3.6.1 SEM

**Fig. 7:**
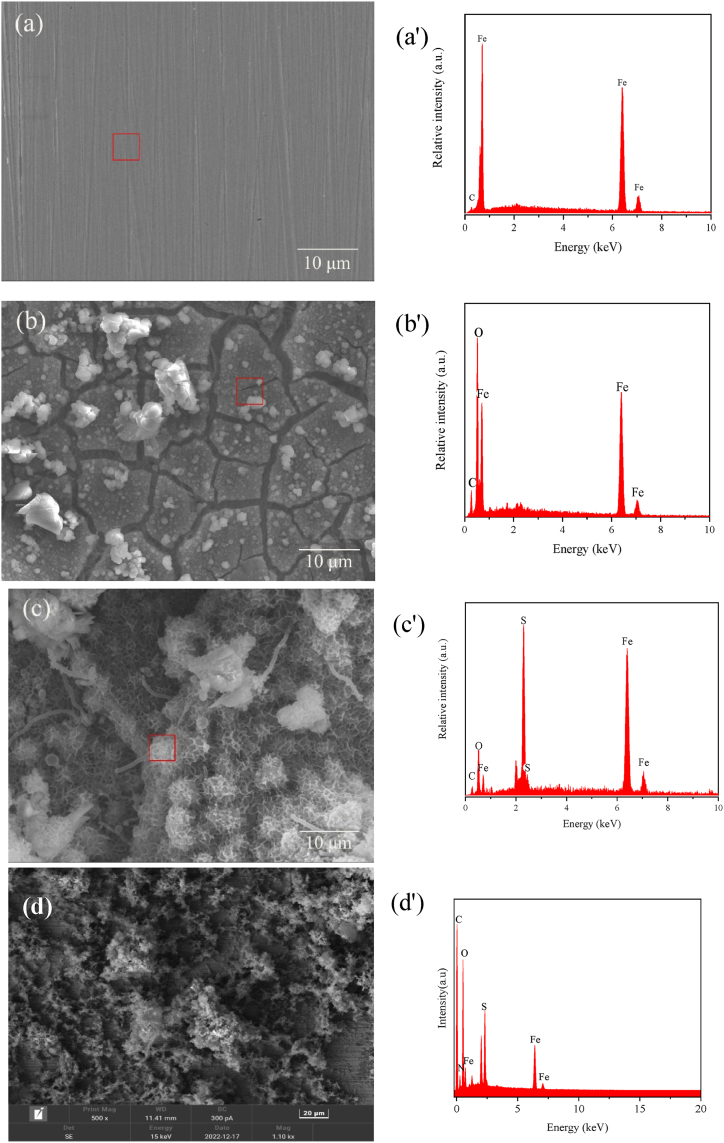

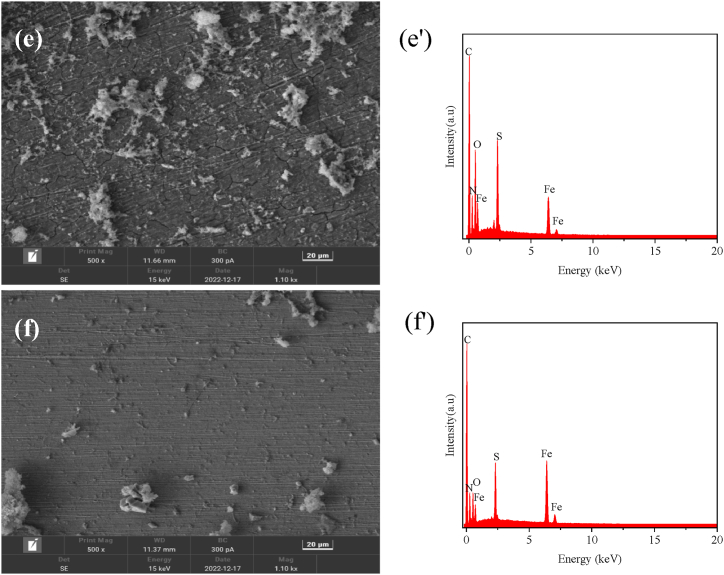
SEM image-EDS data of the surface of carbon steel specimens immersed in different corrosive media for 21 d:(a) carbon steel, (b) sterile, (c) SRB, (d) 0.2 mM 1227, (e) 0.2 mM C_12_-2-C_12_, (f) 0.2 mM C_14_-2-C_14_; and corresponding EDS data (a'-f').

Fig. 7 shows the morphology of corrosion products formed on the surface of carbon steel samples after immersion in SRB solutions with or without 0.2 mM 1227, C_12_-2-C_12_, and C_14_-2-C_14_ for 21 d at 30 °C. The corresponding energy dispersive spectrometer (EDS) analysis results are also presented in Fig. 7.

As shown in Fig. 7 (a), the carbon steel surface is smooth, and the scratches generated by the pre-treatment can be clearly observed. As shown in Fig. 7 (b), there are numerous corrosion products on the surface. As shown in Fig. 7 (c), there are numerous loose products on the surface of the sample, the main component of which is FeS. The SRB cells can be observed in rod-like form, overlapping with the corrosion products. Fig. 7 (d)–7 (f) shows the SEM morphology of the samples after adding 0.2 mM 1227, C_12_-2-C_12_, and C_14_-2-C_14_. The EDS results show that N and S appear on the surface of the sample after adding surfactant. The appearance of S indicates the presence of FeS corrosion products, while the appearance of N indicates that the surfactant can be adsorbed on carbon steel surface. Furthermore, the surface morphology shows that C_14_-2-C_14_ has the highest inhibition efficiency on microbial corrosion.

#### 3.6.2 Atomic force microscopy

AFM was used to further characterize the corrosion morphology of the carbon steel soaked in 0.2 mM 1227, C_12_-2-C_12_, and C_14_-2-C_14_ for 21 d.

**Fig. 8:**
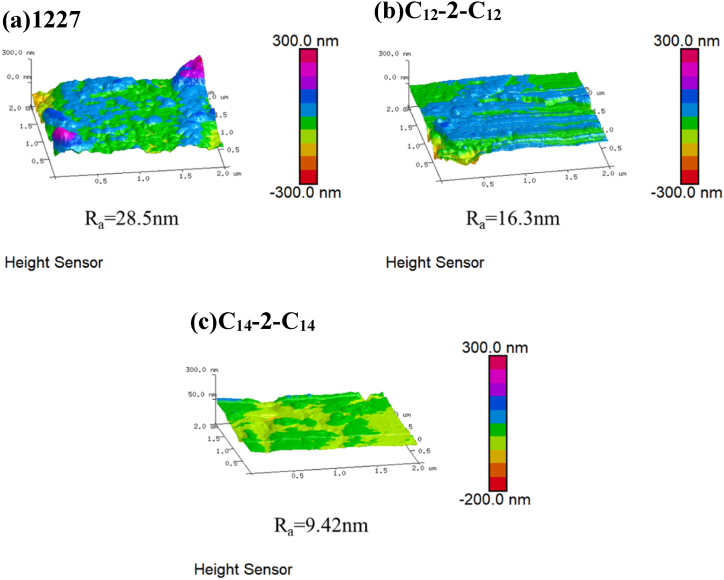
AFM test results of carbon steel specimens after 21d immersion in different media.

As shown in Fig. 8, the average surface roughness (*R*_*a*_) after adding 1227, C_12_-2-C_12_, and C_14_-2-C_14_ is 28.5, 13.6, and 9.42 nm, respectively. The *R*_*a*_ of the samples with C_14_-2-C_14_ is the lowest, indicating that the adsorption film formed on the surface of carbon steel can prevent the erosion of SRB. Herein, the binary quaternary ammonium salt with hydrophobic chain 14 has the best corrosion inhibition and bactericidal properties.

#### 3.6.3 X-ray spectrometer

**Fig. 9:**
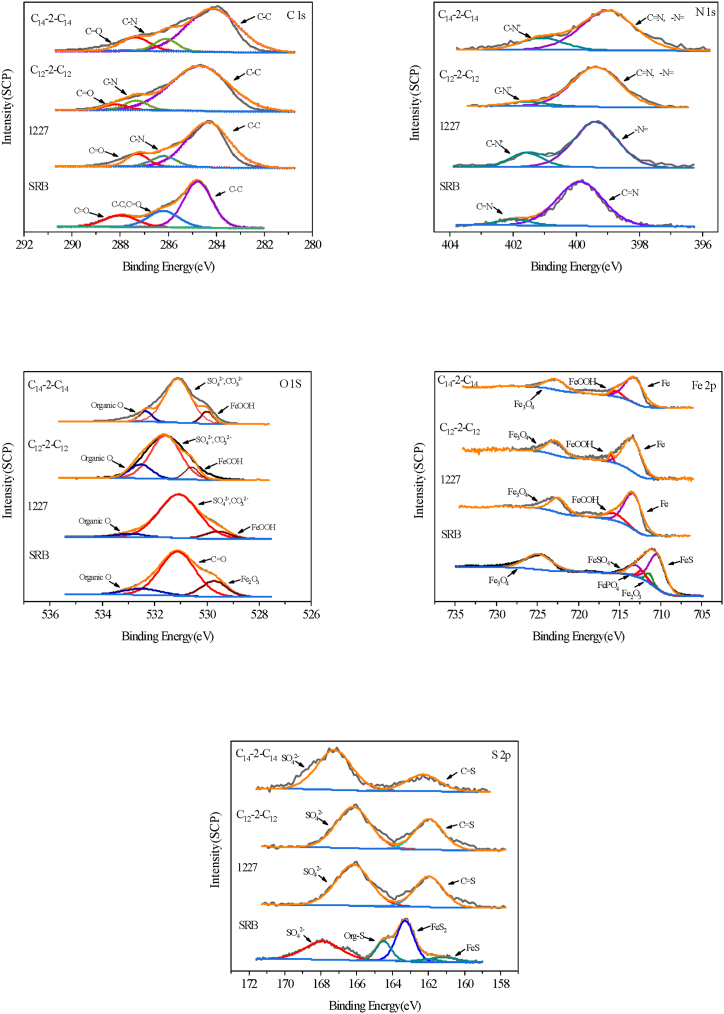
XPS test results of carbon steel specimens after 21d immersion in different media.

Fig. 9 shows the high-resolution XPS spectra of carbon steel surface after 21 d immersion in SRB-containing solution without and with 0.2 mM 1227, C_12_-2-C_12_, and C_14_-2-C_14_. Table 8 presents the peak fitting results.

**Table 8:** Element composition of surface film and XPS fitting parameters of C 1s, O 1s, Fe 2p, S 2p, N 1s after 21 d immersion in different media.

Valence state		Binding energy (eV)	Inverse synthetic bond
C 1s		284.77	C-C
	SRB	286.21	C-C, C=O
		288.00	C=O
		284.38	C-C
	1227	286.08	C-N
		287.48	C=O
		284.68	C-C
	C_12_-2-C_12_	287.38	C-N
		288.28	C=O
		284.18	C-C
	C_14_-2-C_14_	286.08	C-N
		287.28	C=O
O 1s		529.72	Fe_2_O_3_
	SRB	531.11	C=O
		532.50	Organic O
		529.55	FeOOH
	1227	531.15	${\text{SO}}_{4}^{2-},{\text{CO}}_{3}^{2-}$
		532.85	Organic O
		529.58	FeOOH
	C_12_-2-C_12_	531.08	${\text{SO}}_{4}^{2-},{\text{CO}}_{3}^{2-}$
		532.95	Organic O
		528.78	FeOOH
	C_14_-2-C_14_	530.58	${\text{SO}}_{4}^{2-}{,\text{CO}}_{3}^{2-}$
		532.58	Organic O
Fe 2p		710.40	FeS
		711.40	Fe_2_O_3_
	SRB	712.28	FePO4
		713.01	FeSO_4_
		724.84	Fe_3_O_4_
		710.98	Fe
	1227	714.08	FeOOH
		723.78	Fe_3_O_4_
		710.68	Fe
	C_12_-2-C_12_	714.28	FeOOH
		724.08	Fe_3_O_4_
		710.38	Fe
	C_14_-2-C_14_	713.48	FeOOH
Fe 2p		724.28	Fe_3_O_4_
S 2p		161.20	FeS
		162.02	FeS
	SRB	163.30	FeS_2_
		164.50	Org-S
		167.90	${\text{SO}}_{4}^{2-}$
	1227	163.18	C=S
		167.88	${\text{SO}}_{4}^{2-}$
	C_12_-2-C_12_	163.28	C=S
		167.78	${\text{SO}}_{4}^{2-}$
	C_14_-2-C_14_	162.88	C=S
		167.68	${\text{SO}}_{4}^{2-}$
N 1s	SRB	399.87	C=N
		402.00	C=N
	1227	399.38	-N =
		401.58	C-N^+^
	C_12_-2-C_12_	399.18	C=N, -N =
		402.08	C-N^+^
	C_14_-2-C_14_	398.98	C=N, -N =
		401.18	C-N^+^

As shown in Fig. 9, the carbon steel surface submerged in the SRB solution has only C–C and C=O peaks presented in the C1s spectrum, suggesting that the surface has undergone oxidation. However, when adding 1227, C_12_-2-C_12_, and C_14_-2-C_14_, a C–N peak appears on the carbon steel surface, proving that the surfactants have been adsorbed on the carbon steel surface. According to the fitting results of the Fe2p spectrum, the primary peaks in the SRB medium are FeS, Fe_2_O_3_, FePO_4_, FeSO_4_, and Fe_3_O_4_, with the binding energies being 710.40, 711.40, 712.38, 713.01, and 724.84 eV, respectively. The appearance of iron oxides indicates the generation of passivation films, while the FeOOH peak mainly occurs in the SRB solution with surfactant.

According to the S2p spectrum, the main component of corrosion products in the SRB medium is FeS, and the emergence of organic sulfur can be attributed to proteins and related compounds produced by bacteria [57,58]. Meanwhile, the generation of FeS and FeS_2_ indicates that the metabolic activities of SRB lead to the conversion of iron oxides to FeS. After adding 1227, C_12_-2-C_12_, and C_14_-2-C_14_, only C=S and SO_4_^2−^ appear on the carbon steel surface. Combining the spectral results, it can be concluded that surfactant molecules can prevent the contact between carbon steel and SRB by adsorbing on the surface of carbon steel, consequently slowing down corrosion.

### 3.7 MD simulation

MD simulation is a method used to study the interaction between surfactant molecules and the surface of carbon steel. This study investigated the main and top views of the dynamic adsorption simulation of C_12_-2-C_12_ and C_14_-2-C_14_ on the surface of carbon steel.

**Fig. 10:**
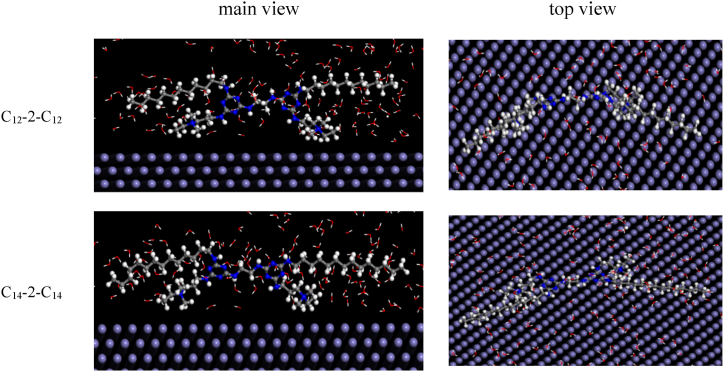
Molecular dynamics simulation diagram of two surfactant molecules.

Fig. 10 shows the equilibrium adsorption configuration of surfactant molecules on the Fe (110) surface. It can be seen that the corrosion inhibitor molecules are adsorbed on the Fe (110) surface in parallel after the system reaches equilibrium. This is because the surfactant molecules contain triazine rings and N atoms, providing more electrons for the d orbitals of Fe.

The adsorption energy of surfactant molecules on the iron surface was calculated from Eq. (7) [59]. The results are presented in Table 9.

(7) $$E_{\text{ads}}=E_{\text{total}}-\left(E_{\text{sur}}+E_{\text{sur}+\text{water}}\right)$$

where *E*_ads_ is the binding energy, *E*_sur_ is the energy of the corrosion inhibitor molecule, *E*_total_ is the total energy of the whole system, and *E*_sur+water_ is the total energy of metal surfaces and water molecules without inhibitors. The value of the binding energy, *E*_binding_, is equal to the opposite value of *E*_ads_ [60]:

(8) $$E_{\text{bingding}}=-E_{\text{ads}}$$

**Table 9:** Molecular dynamics related parameters of two surfactants.

Inhibitor	*E*_total_ (kJ mol^−1^)	*E*_sur_ (kJ mol^−1^)	*E*_binding_ (kJ mol^−1^)	*E*_ads_ (kJ mol^−1^)
C_12_-2-C_12_	−20572.121	−1640.542	832.469	−832.469
C_14_-2-C_14_	−21413.716	−1399.628	2450.802	−2450.802

The binding energy reflects the adsorption stability of the inhibitor on the iron surface. As can be seen from the data presented in Table 9, the binding energies of C_12_-2-C_12_ and C_14_-2-C_14_ with Fe are 832.469 and 2450.802 kJ mol^−1^, respectively. The higher binding energy indicates that the surfactant is more easily adsorbed to the carbon steel surface, indicating a better corrosion inhibition performance. Therefore, the corrosion inhibition effect of C_14_-2-C_14_ is greater than that of C_12_-2-C_12_.

### 3.8 Mechanism analysis

**Fig. 11:**
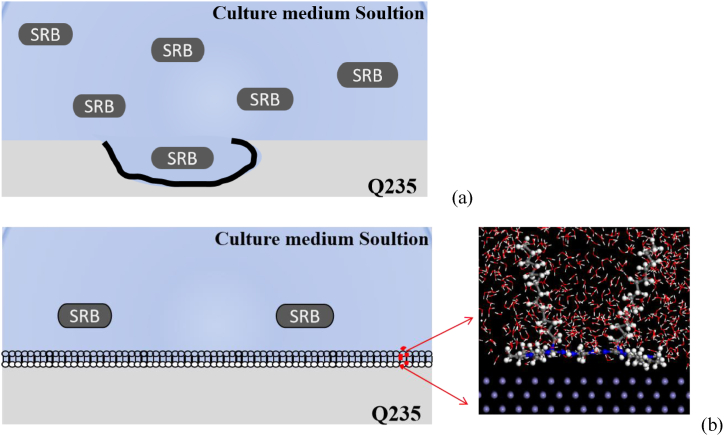
Schematic diagram of adsorption behavior of metal surface corrosion inhibitor molecules in the medium solution.

In the SRB solution (Fig. 11 (a)), SRB is involved in the electron transfer process and produces S^2−^, HS^−^ or some organic acids in the metabolic process, which can cause or accelerate the corrosion of carbon steel under anaerobic conditions. Meanwhile, SRB metabolites react with the carbon steel to produce FeS, which reduces the smoothness of the carbon steel surface, facilitates the attachment of SRB and extracellular cell transfer, and promotes the dissolution, thus accelerating corrosion. After the addition of surfactant (Fig. 11 (b)), the surfactant molecules can be adsorbed on the carbon steel surface to form a dense adsorption film, thus hindering the erosion of SRB on the carbon steel. Meanwhile, the positive charge N^+^ in the surfactant molecules interacts with the negative charge on the cell wall of SRB, and the hydrophobic chain ends fuse with the cell membrane, which can block the metabolism of SRB and led to the death of SRB, thus slowing down the corrosion.

## 4 Conclusion

(1) The minimum inhibitory concentrations of 1227, C_12_-2-C_12_, and C_14_-2-C_14_ are 0.300, 0.021, and 0.005 mM, respectively.
(2) The results reveal that 0.2-mM 1227, C_12_-2-C_12_, and C_14_-2-C_14_ lead to corrosion inhibition rates of 76.49 %, 88.45 %, and 93.23 %, respectively. This indicates that all three surfactants can effectively suppress the corrosion of carbon steel in the SRB solution. Moreover, C_14_-2-C_14_ has the best inhibition effect, further indicating that the Gemini surfactant with a hydrophobic chain of 14 carbon atoms has better corrosion inhibition and sterilization performance in the investigated range.
(3) SEM and AFM results show that 1227, C_12_-2-C_12_ and C_14_-2-C_14_ are excellent fungicides, with C_14_-2-C_14_ having the best antibacterial effect. The EDS and XPS surface analysis results indicate that the surfactant molecular can adsorb on the surface of carbon steel, inhibiting the SRB corrosion.

## CRediT authorship contribution statement

**Guofang Gao:** Writing – original draft, Investigation. **Junxia Wang:** Software, Investigation. **Penghui Liang:** Formal analysis. **Yilei Ruan:** Data curation. **Dehua Wang:** Data curation. **Li Feng:** Conceptualization. **Xuemei Ma:** Methodology. **Zhiyong Hu:** Project administration. **Hailin Zhu:** Writing – review & editing, Supervision, Conceptualization.

## Data availability statement

The authors declare that the data will be available from the corresponding author under reasonable request.

## Funding

This work was supported by Fundamental research program of Shanxi Province (No. 202103021224207, 20210302124337) and the 19th graduate science and technology project of 10.13039/501100007926North University of China (20231926).

## Declaration of competing interest

The authors declare the following financial interests/personal relationships which may be considered as potential competing interests: Fundamental research program of Shanxi Province (No.202103021224207, 20210302124337)The 19th graduate science and technology project of North University of China (20231926)
