# Supplementary material for: Two novel triazine-based quaternary ammonium salt Gemini surfactants as potential corrosion inhibitors for carbon steel in a sulfate-reducing bacteria solution: Experimental and theoretical studies
Source: Heliyon. 2024 Nov 20;10(23):e40385. doi: 10.1016/j.heliyon.2024.e40385 (PMC11629201; doi:10.1016/j.heliyon.2024.e40385)
Supplement: Multimedia component 1 [file mmc1.docx]

**Two novel triazine-based quaternary ammonium salt Gemini surfactants as potential corrosion inhibitors for carbon steel in a sulfate-reducing bacteria solution: experimental and theoretical studies**

Guofang Gao^1,2^, Junxia Wang^1,2^, Penghui Liang^1,2^, Yilei Ruan^1,2^, Dehua Wang^1,2^, Li Feng^1,2^, Xuemei Ma^1,2^, Zhiyong Hu^1,2^, Hailin Zhu^1,2^[[1]](#footnote-0)^ *^

(1 *School of Chemistry and Chemical Engineering, North University of China, Taiyuan, 030051, Shanxi, China; 2 Shanxi Key Laboratory of Functional Surfactants, Taiyuan 030001, Shanxi, China*)

1. **Characterization for C_14_-2-C_14_**


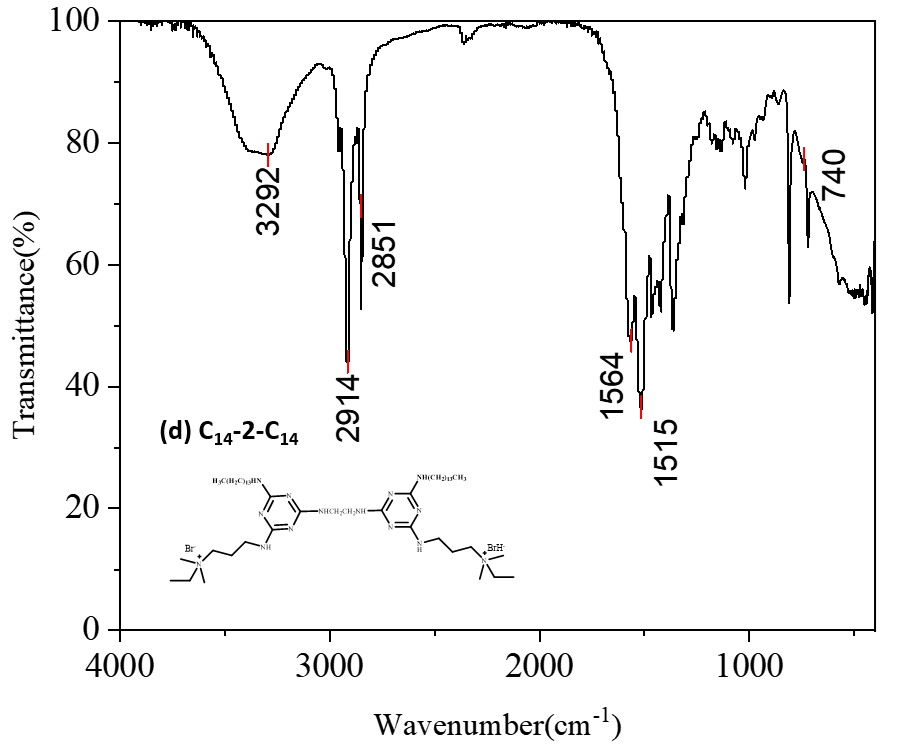


Fig. S1. FT-IR images of C_14_-2-C_14_.

| Table S1  Characteristic absorption peak assignment of C_14_-2-C_14_. |
| --- |
| \| FT-IR peak \| C_14_-2-C_14_ (cm^-1^) \| \| --- \| --- \| \| N-H stretching vibration peak \| 3292 \| \| Methyl C-H stretching vibration peak \| 2914 \| \| Methylene C-H stretching vibration peak \| 2851 \| \| Triazine ring C=N \| 1564 \| \| Telescopic vibration peak \| 1515 \| \| -(CH_2_)_n_-in-plane rocking vibration peak \| 740 \| |

| 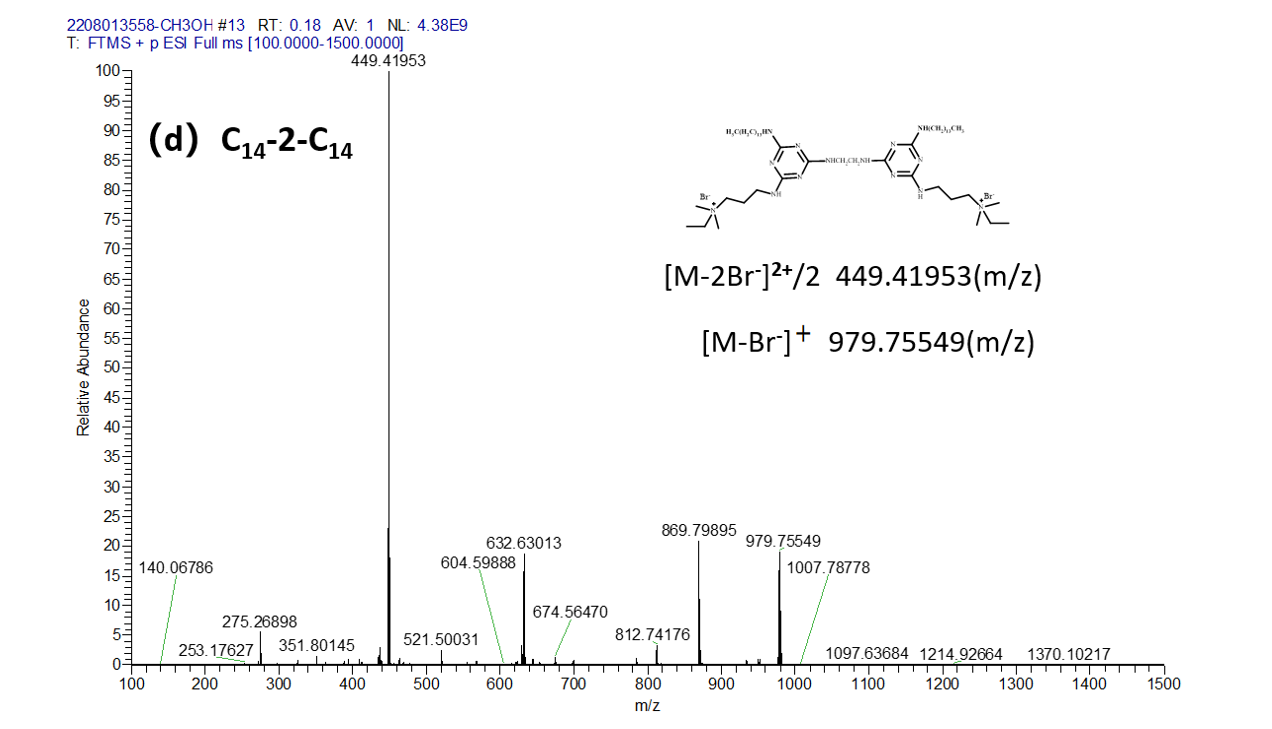 |
| --- |
| Fig. S2. ESI-MS spectra of C_14_-2-C_14_ |

| Table S2  Mass spectrometry data of C_14_-2-C_14_. |
| --- |
| \| Surfactant \| Structural formula \| Molecular formula \| [M-2Br^-^]^2+^/2 \| [M-Br^-^]^+^ \| \| --- \| --- \| --- \| --- \| --- \| \| C_14_-2-C_14_ \|  \| C_50_H_102_Br_2_N_14_ \| 449.41953 \| 979.75549 \| |

| 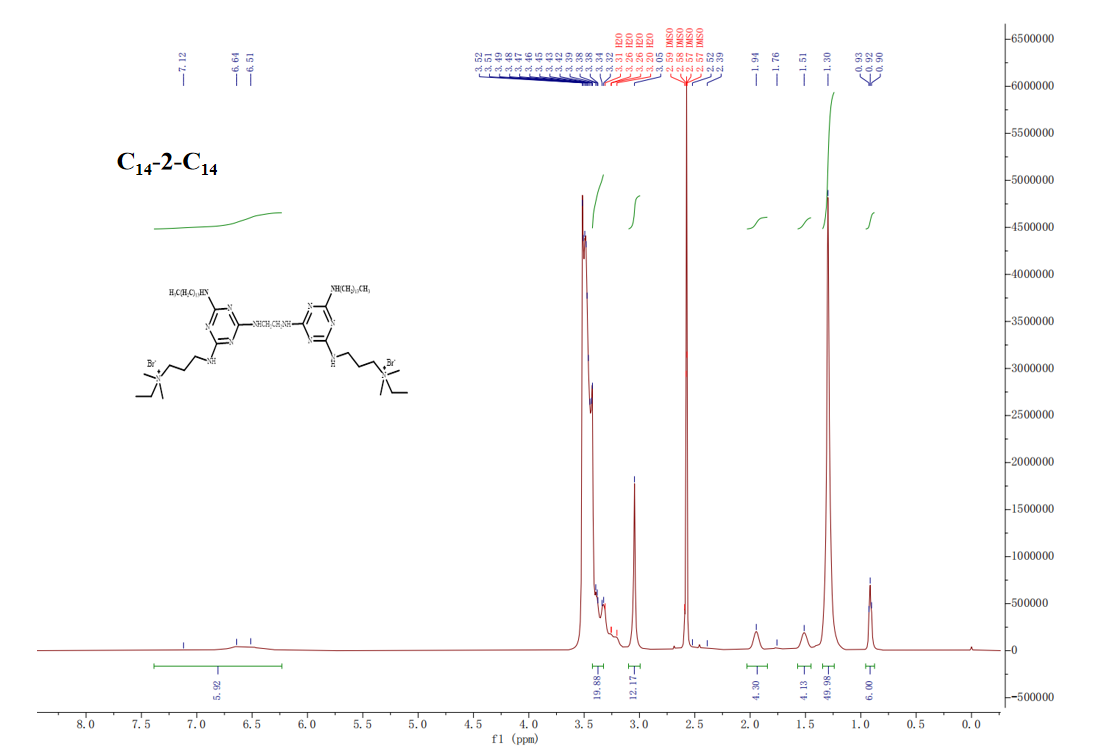 |
| --- |
| Fig. S3. ^1^H NMR spectra of C_14_-2-C_14_. |

| Table S3  ^1^H NMR spectrum results of C_14_-2-C_14_. |
| --- |
| \| Surfactant \| ^1^H NMR δ/ppm \| \| --- \| --- \| \| C_14_-2-C_14_ \| 0.903-0.927 (t, C**H**_3_-CH_2_-CH_2_-, 6H), 1.297 (m, CH_3_-(C**H**_2_)_11_-CH_2_-CH_2_, 36H) & (s, -NH-CH_2_-C**H**_3_, 6H), 1.512(d, -NH-CH_2_-C**H**_2_-, 4H), 1.945 (s, (CH_3_)_2_-N-CH_2_-C**H**_2_-, 4H), 3.045 (t, (C**H**_3_)_2_-N-CH_2_-, 12H), 3.324-3.935 (m, -C**H**_2_-NH-, 20H), 6.512-7.119 (d, C-N**H**-, 6H) \| |

**2. Growth curve of SRB**

|  |
| --- |
| Fig. S4. Growth curve of SRB in medium solution. |

**3. Surface activity**

In order to study the surface activity of two synthesized quaternary ammonium salt Gemini surfactants containing triazine rings in 3.5% NaCl solution, the surface tension of the two surfactants was measured by surface tension method as a function of concentration *γ*-lg*C* curve is shown in Fig. S5.

|  |
| --- |
| Fig. S5. *γ*-lg*C* curves of bis-quaternary ammonium salt surfactant containing triazine group in 3.5% NaCl. |

The surface pressure (*π*_cmc_), saturation adsorption capacity (*Γ*_max_), limiting molecular area (*A*_min_) and p*C*_20_ of the four surfactants were calculated according to equations 1 to 4, and the corresponding results were listed in Table 4.

$\pi_{cmc}=\gamma_{0}-\gamma{}_{_{cmc}}$ (1)

$\Gamma_{\max}=\left( \frac{-1}{2.303nRT} \right)\cdot\left( \frac{d\gamma}{\mathrm{dlg}C} \right)_{T}$ (2)

$A_{\min}=\frac{10^{14}}{N_{A}\Gamma_{\max}}$ (3)

$pC_{20}=\frac{\gamma_{0}-20-\gamma_{\mathrm{cmc}}}{2.303nRT\Gamma_{\max}}-\frac{ln\mathrm{cmc}}{2.303}$ (4)

In the formula, *γ*_0_ is the surface tension value of 3.5% NaCl solution (70.40 mN/m), *γ*_cmc_ is the surface tension value of the solution corresponding to the critical micelle concentration of the Gemini surfactant containing triazine cyclic quaternary ammonium salt, mN/m; *n* is the number of solute types in the surfactant solution, for 1:1 ionic surfactants and containing an excess of electrolytes with common counter ions, n=1; *R* is the gas constant, 8.314 J/(mol·k); *T* is the absolute temperature, K; $\frac{d\gamma}{\mathrm{dlg}C}$is the slope of the logarithmic curve of surface tension and concentration; *C* is the concentration of the surfactant, M; *N*_A_ is Avogadro's constant, 6.02×10^23^ mol^-1^.

| Table S4  Surface activity data. |
| --- |
| \| Inhibitor \| cmc  (mM) \| *γ* _cmc_  (mN/m) \| *π*_cmc_  (mN/m) \| *Γ*_max_×10^10^  (mol/cm^2^) \| *A*_min_  (nm^2^) \| p*C*_20_ \| \| --- \| --- \| --- \| --- \| --- \| --- \| --- \| \| C_12_-2-C_12_ \| 0.06 \| 43.39 \| 26.91 \| 1.91 \| 0.87 \| 1.26 \| \| C_14_-2-C_14_ \| 0.01 \| 47.38 \| 22.92 \| 2.23 \| 0.90 \| 2.05 \| |

As can be seen from Fig. S5, surface tension of C_12_-2-C_12_ and C_14_-2-C_14_ first decreases and then remains unchanged with the increase of surfactant concentration. From Table S4, the cmc values of the two surfactants are 0.06 mM and 0.01 mM, respectively, and the critical micelle concentration of C_14_-2-C_14_ is lower. This is mainly because the affinity for micelle formation increases with the increase of the length of the hydrophobic chain, which effectively reduces the critical micelle concentration of 3.5% NaCl solution of the surfactant. The p*C*_20_ value can be used to measure the efficiency of surfactants in reducing surface tension, and the higher the p*C*_20_ value is, the higher the adsorption efficiency of surfactants at the interface. As can be seen from Table 4, the p*C*_20_ value of C_14_-2-C_14_ is higher than that of C_12_-2-C_12_, indicating that C_14_-2-C_14_ has higher adsorption efficiency.

**4. Equivalent circuit**

| 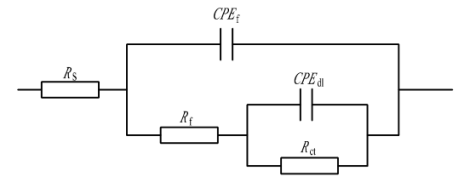 | 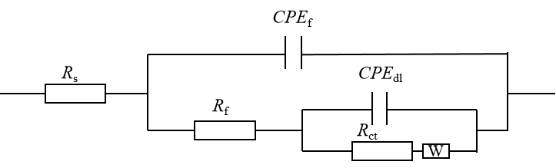 |
| --- | --- |
| (a) | (b) |
| Fig. S6. Equivalent circuit diagram. | |

1. Correspondence: Hailin Zhu

   Email: zhuhailin@nuc.edu.cn, zhuhailin99@126.com [↑](#footnote-ref-0)
